# Serial Skin Grafing Technique for Management of a Facial Giant Congenital Melanocytic Nevus: Case Report with a Literature Review

**DOI:** 10.1055/s-0045-1806754

**Published:** 2025-03-13

**Authors:** Anna Chrapusta, Marek Kachnic, Anna Jurczyszyn, Maria Kamila Klimeczek-Chrapusta

**Affiliations:** 1 Malopolska Burn and Plastic Surgery Center, Ludwik Rydygier Memorial Hospital in Krakow, Cracow, Poland; 2 Department of Pediatric Surgery, Faculty of Medicine, Jagiellonian University Medical College, Cracow, Poland; 3 Faculty of Medicine, Jagiellonian University Medical College, Cracow, Poland

**Keywords:** facial reconstruction, melanocytic nevus, pediatric surgery

## Abstract

We present a case report of a 6-month-old infant with a giant congenital melanocytic nevus (GCMN) covering 50% of the face. The treatment of choice was a serial resection with skin grafting. The first surgery encompassed excision from the nose and the right and left infraorbital areas. Full-thickness skin graft (FTSG) harvested from the left inner arm was engrafted. During the second surgery, the GCMN was excised from the left cheek, which was reconstructed with a fasciocervical advancement flap elevated in a superomedial direction. The third surgery encompassed excision from the forehead, left temporal region, right cheek, and left lower eyelid. The area was covered with an FTSG from the right inner brachium. At the age of 3 years, resection from the left eyelid, skin grafting, and temporal tarsorrhaphy were performed. The orbicularis oculi were released 1.5 years later and the lower eyelid was reconstructed with a fragment of a cartilage graft from the auricular conchae. Following the patient's complete healing, their quality of life was evaluated through Pediatric Quality of Life Inventory report for toddlers. A pleasant aesthetic result was achieved, satisfying both parents and the child.

## Introduction

A congenital melanocytic nevus (CMN) is a dermatological phenomenon characterized by melanocytic, hairy, noncancerous patches of skin, present at birth or developing shortly thereafter. 1 CMNs are classified according to the size that they are predicted to reach in adulthood (projected adult size [PAS]). Giant CMNs (GCMNs; >40 cm PAS) occur in approximately 1/500,000 newborns worldwide. 2 GCMNs sometimes occur along with multiple smaller melanocytic nevi, called “satellite nevi.” 1 2

While typically benign in nature, GCMNs harbor a risk of malignant transformation into melanoma. 3 The risk of melanoma appears proportional to the lesion size, reaching up to 10 to 15% risk of a lesion greater than 40 cm in diameter. These patients are also recommended to undergo MRI screening for deposits of melanocytic cells along the leptomeninges, called neural melanosis, which can be asymptomatic or present with seizures or developmental delay. 2 3 4

We present the case of a 6-month-old infant with a GCMN of more than 40 cm PAS, which covered over 50% of the face. The purpose of this case report is to describe and assess our treatment outcomes as well as highlight the advantages of a multistep surgical approach compared to other management strategies.

## Case Report

**Fig. 1:**
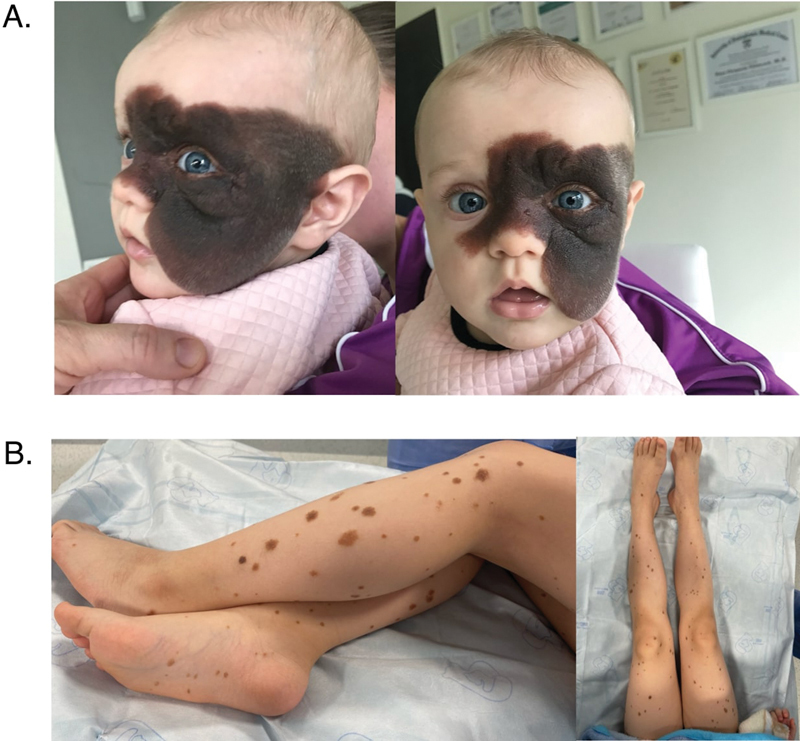
Pictures taken during an initial visit when the patient was 8 months old. ( **A** ) Facial giant congenital melanocytic nevus (GCMN). ( **B** ) Satellite nevi on lower limbs.

**Fig. 2:**
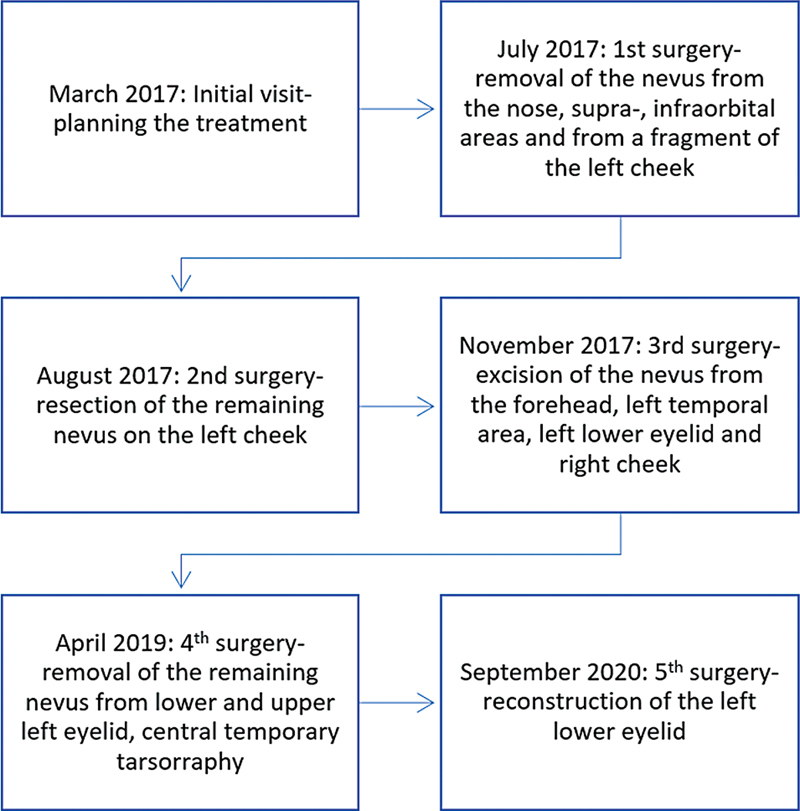
Timeline presenting the surgeries performed.

A 6-month-old infant was presented to a plastic surgery clinic with a dark and hirsute GCMN covering approximately 50% of the face, including the entire left cheek, left eyelid, supra- and infraorbital region, nasal bridge, and forehead. “Satellite nevi” were recognized on the patient's trunk and both upper and lower limbs ( Fig. 1 ). No other anomalies were detected. The patient did not undergo genetic testing for possible mutations. During the initial visit, a multistep surgical approach was planned ( Fig. 2 ).

The first surgery took place when the patient was 11 months old, beginning with a partial excision of the nevus from the part of the left supra- and infraorbital area, nasal bridge, and a fragment of the left cheek. The tissue of the nevus was sent out for a histopathological evaluation. A full-thickness skin graft (FTSG) was taken from the left arm and transplanted onto that area to cover the tissue deficiency. The left arm was closed primarily.

**Fig. 3:**
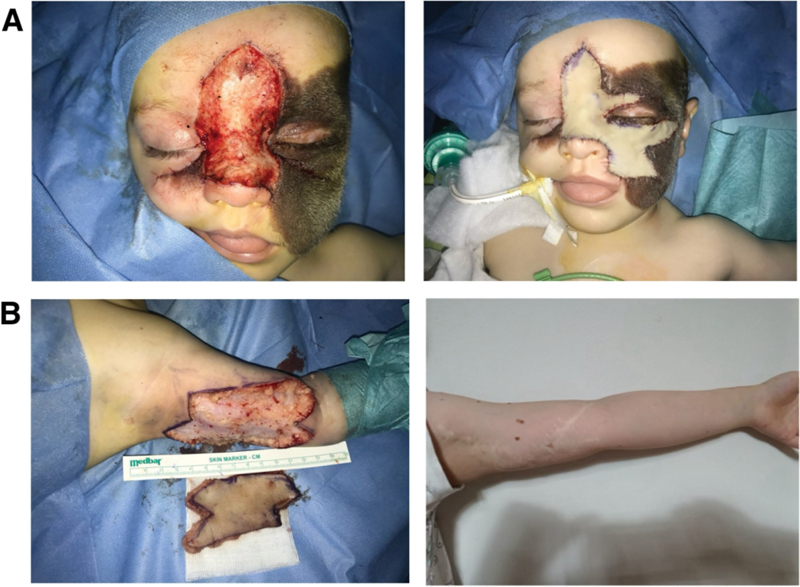
( **A** ) A portion of the nevus was surgically removed from the nose, supraorbital and infraorbital areas, and a fragment of the left cheek. A full-thickness skin graft (FTSG) was taken from the left arm and transplanted onto the face. The left arm was then closed primarily. ( **B** ) Healed area from which FTSGs were being harvested.

In all the surgeries, the inner part of the arm was consistently chosen as the donor site for harvesting FTSGs ( Fig. 3 ). FTSGs were harvested twice from the left inner arm and once from the right inner arm. The largest graft measured 10 × 5.5 cm. To achieve optimal aesthetic outcomes at the donor sites, a wide dissection of the adipocutaneous flaps was performed to facilitate primary closure. Postoperative scar management included the use of wound closure strips, which effectively approximated the wound edges, contributing to cosmetically favorable results.

**Fig. 4:**
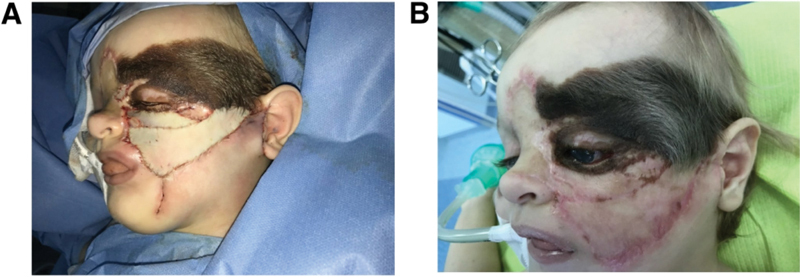
( **A** ) Further resection of the nevus from the left cheek. Cervicofascial flap was advanced from the mandibular region, where a cutaneous flap was elevated from the platysma and moved in the superomedial direction to cover the skin deficiency. The medial part of the cheek was reconstructed with a full-thickness skin graft (FTSG) harvested from the right arm. ( **B** ) Healed operated area.

The second surgery occurred 4 weeks later, encompassing further excision of the nevus from the left cheek. The technique for this surgery was a cervicofascial flap advanced from the mandibular region, where a cutaneous flap was elevated from the platysma and moved in the superomedial direction to cover the skin deficiency. In order to avoid tension, the medial part of the cheek was reconstructed with an FTSG harvested from the right arm, which was closed primarily ( Fig. 4 ).

Four months later, another resection was done to address the nevus on the forehead, left temporal region, left lower eyelid, and right cheek. An FTSG taken from the left arm was engrafted into the mentioned areas.

**Fig. 5:**
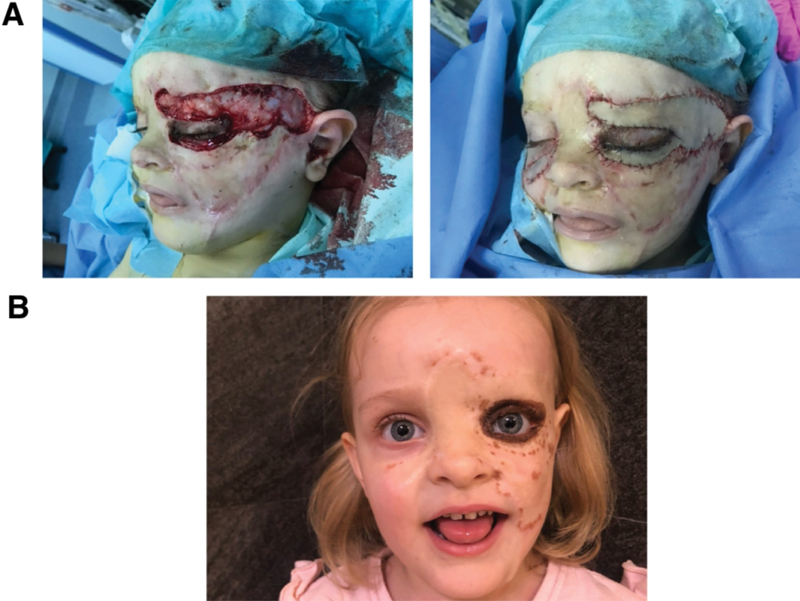
( **A** ) Resection of the remaining nevi within the margins of the postoperative scars was executed. Central temporary tarsorrhaphy was done to ensure proper healing of the eyelids. ( **B** ) Results after almost a year after surgery.

At the age of 3 years, a surgical procedure targeted the nevus within the left lower and upper eyelids, coupled with a reconstruction, that used an FTSG from the left arm. Central temporary tarsorrhaphy was done to ensure proper healing. Resection of the remaining nevi within the margins of the postoperative scars was executed ( Fig. 5 ).

**Fig. 6:**
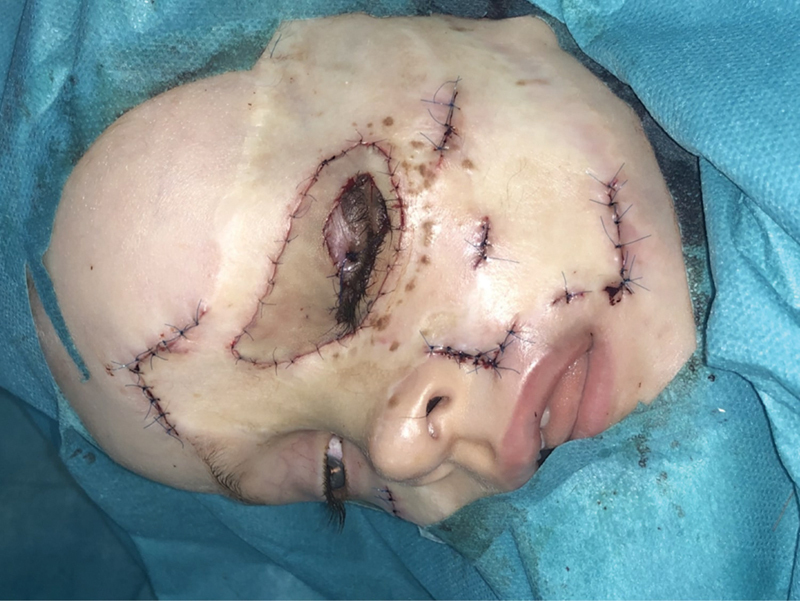
Reconstruction of the left lower eyelid with a cartilage harvested from the left auricle with excision of the remaining nevi within the margins of the scars.

Reconstruction of the left lower eyelid with a cartilage from the left auricle was done 1.5 years later with a lowering of the palpebral margin. The auricular cartilage was used to reconstruct the tarsal plate of the lower eyelid. Skin defects resulting from the nevus excision were covered with an FTSG from the area posterior to the right auricle ( Fig. 6 ).

**Fig. 7:**
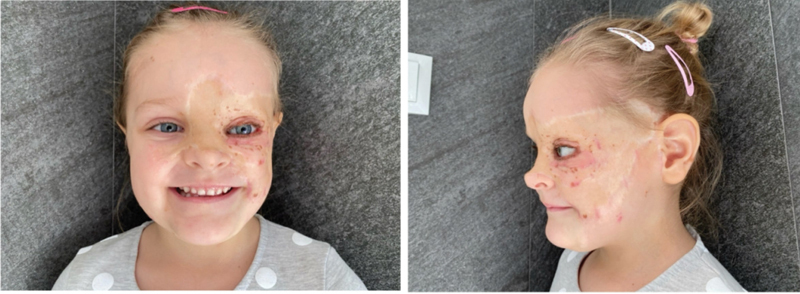
Most recent pictures taken of our patient.

This patient remains under the plastic surgeon's observation. In the resected areas, no signs of reappearance of the nevus were detected; therefore, it was deemed successful. Genetic screening for NRAS and BRAF mutations was advised ( Fig. 7 ).

The overall quality of life and functional and aesthetic outcomes were evaluated 2 years after the last surgery. We used the Pediatric Quality of Life Inventory (PedsQL) parent and patient report for young children aged 5 to 7 years, which is a subjective questionnaire filled in by parents and their child separately. Total scores of 84.4 for the parent report and 83.1 for child report were achieved, which are considered highly satisfactory and mean a high health-related quality of life (HRQL). Table 1 depicts the results.

**Table 1:** Results of the Pediatric Quality of Life Inventory Generic Core for young children aged 5 to 7 years questionnaire

Dimensions	Number of items	Score for parent report	Score for child report
Physical functioning	8	87.5	87.5
Emotional functioning	5	90	90
Social functioning	5	80	80
School functioning	5/6	80	75
Total	23	84.4	83.1

Note: The questionnaire evaluated four dimensions of the patient's life in the past month. Scores are presented on the scale of 0 to 100. The higher the score, the better the health-related quality of life.

## Discussion

This study describes a technique for management of a facial GCMN with the use of a fascio-cervical flap, serial excision, and multiple FTSGs harvested from an inner part of the arm. Surgical excision of a GCMN requires careful consideration of several factors, including malignancy risk, cosmetic outcomes, psychological impact, and the risks associated with repeated general anesthesia.

The inner part of the arm serves as an optimal donor site for FTSGs due to small skin tensioning, substantial subcutaneous adipose layer that enables primary closure, and very small density of hair follicles that tend to be lighter in color and similar shade to the skin of the patients face. Moreover, this body area is usually hidden and is less vulnerable to sun exposure, which is important regarding the scarring process. Skin in that region is also thinner, so when harvested properly it does not require additional thinning, which reduces the operative time. 5

Our case report details a novel surgical approach for this type of lesion. The uniqueness of our case lies in the novel approach to this type of reconstruction. In this patient, we did not use tissue expanders and a large part of the tissue defect was covered by advancing a fasciocutaneous cervicofacial flap. Moreover, it is unique because the patient was 1 year old at the time of the surgery. The uniqueness of our case is the lesion itself as it is very rare for a child to be born with a GCMN covering over 40 cm of their face. A nonstandard method of treating the donor site scar (with consistent tight taping with wound closure strips, placed under tension to bring the ridges of the scar together) allowed for the harvesting of another large FTSG from the same site just 6 months later.

Our case report presents a novel surgical approach to addressing this type of lesion. The uniqueness of this case lies in the employed reconstruction method. Notably, tissue expanders were not used; instead, a significant portion of the tissue defect was covered by advancing a fasciocutaneous cervicofacial flap. Additionally, the surgery was performed on a 1-year-old child. The rarity of the lesion itself further underscores the distinctiveness of this report. Furthermore, a nonstandard method of managing the donor site scar—utilizing consistent, tight taping with wound closure strips applied under tension to approximate the scar edges—enabled the successful harvesting of another large FTSG from the same site just 6 months later.

Cheng et al 1 discovered that surgical excision led to shorter postoperative hospitalization, quicker recovery, and lower number of complications in comparison with tissue expanding or skin grafting. Tissue expanders were associated with higher psychological burden, lasting up to 6 months, and increased pain, frequent office visits, and functional disability in children. 1 Rotation or transposition of expanded flaps creates longer and more visible scars when compared to linear scars from serial excision. 6 7 In our case, a single-step tissue expansion or serial excision without skin grafting cannot address the entire defect. 8 Kim et al, 6 in a review of 88 medical records, compared single and serial expansion techniques for such lesions and concluded that infection and revisional operation rates were significantly higher in the serial expansion groups.

While the literature underscores the advantages of an early surgical intervention, consensus regarding the optimal age for excision remains a subject of ongoing debate. 9 We began treatment at a very young age, because of the psychological burden of a GCMN that was distorting the child's appearance and also affected the parent's well-being. As proper psychosocial functioning is at high stake, several different studies point toward the importance of cosmetic outcomes. Koot et al 10 measured the psychosocial functioning of 29 children affected by GCMNs. Thirty percent of them scored under the 5th percentile, particularly in social competence. The children used to avoid situations when they would have to undress or uncover the affected body parts and 70% of mothers reported that they found it grueling and hard to accept having a child with a GCMN. 4 In this case report, scores of 84.4 for the parent report and 83.1 for the child report were achieved using the PedsQL. Our results are satisfactory in comparison to the literature, especially considering that our patient struggled with a facial GCMN.

Another reason behind early intervention is the risk of developing melanoma. Scard et al, 11 in their systematic review on the risk of melanoma in melanocytic nevi of all sizes, report that the median age of melanoma onset is 22 years for patients with small- and medium-sized CMNs, compared to 9.5 years for those with large CMNs or GCMNs. Thus, treatment aims at lowering the risk of metastasis in the future. The incidence of melanoma after 15 years was 0.132% in patients who received partial treatment (partial surgery, laser, dermabrasion, curettage, or peelings), while no patients who underwent complete excision developed melanoma within the 15-year follow-up period. 11

The limitations of our treatment lie in the unknown etiology of the nevus, due to the absence of the genetic testing provided by the patient. Regrowth of the nevus around the palpebral margin was observed, which was why the patient was qualified for another surgery. Fortunately, no signs of regrowth were observed in other excised areas even after almost 7 years since surgery.

## Conclusion

In conclusion, while FTSGs rank lower than tissue expansion on the reconstructive ladder, this case highlights their successful use, when combined with local tissue transfer for the complete excision of a GCMN in challenging areas such as the face. To our knowledge, this is the first report of this technique with a follow-up and quality of life assessment. Wide suprafascial dissection of the arm tissues allows for the harvesting of exceptionally large skin grafts with primary wound closure. Selecting the inner arm as the donor site seems to be a promising solution for achieving good aesthetic results.

## References

[1] Cheng X Wang W He Y Bao W Jia C Dai T Comparison of three different surgical approaches for the treatment of large/giant congenital melanocytic nevus J Cosmet Dermatol 2022 21 10 4609 4616 35810351 10.1111/jocd.15219

[2] Jahnke M N O'Haver J Gupta D Care of congenital melanocytic nevi in newborns and infants: review and management recommendations Pediatrics 2021 148 06 e2021051536 34845496 10.1542/peds.2021-051536

[3] Viana A C Gontijo B Bittencourt F V Giant congenital melanocytic nevus [published correction appears in An Bras Dermatol. 2014 Jan-Feb;89(1):190] An Bras Dermatol 2013 88 06 863 878 24474093 10.1590/abd1806-4841.20132233 PMC3900335

[4] Charbel C Fontaine R H Malouf G G NRAS mutation is the sole recurrent somatic mutation in large congenital melanocytic nevi J Invest Dermatol 2014 134 04 1067 1074 24129063 10.1038/jid.2013.429

[5] Osman O F Emara S Extended use of full-thickness skin grafts, employing variable donor sites World J Plast Surg 2018 7 02 159 165 30083497 PMC6066711

[6] Kim M J Lee D H Park D H Multifactorial analysis of the surgical outcomes of giant congenital melanocytic nevi: single versus serial tissue expansion Arch Plast Surg 2020 47 06 551 558 33238342 10.5999/aps.2020.01494 PMC7700868

[7] Hassanein A H Rogers G F Greene A K Management of challenging congenital melanocytic nevi: outcomes study of serial excision J Pediatr Surg 2015 50 04 613 616 25840073 10.1016/j.jpedsurg.2014.08.020

[8] Sơn T T Nghĩa P T Dung P TV Thuý T TH Anh H T Huy L A Serial tissue expansion and skin grafts in the management of a giant congenital nevus of the face: review of literature and case report Arch Plast Surg 2024 51 03 290 294 38737851 10.1055/a-2201-8061 PMC11081729

[9] Sawicka E Szczygielski O Żak K Pęczkowski P Michalak E Bekiesińska-Figatowska M Giant congenital melanocytic nevi: selected aspects of diagnostics and treatment Med Sci Monit 2015 21 123 132 25577155 10.12659/MSM.891279 PMC4298998

[10] Koot H M de Waard-van der Spek F Peer C D Mulder P G Oranje A P Psychosocial sequelae in 29 children with giant congenital melanocytic naevi Clin Exp Dermatol 2000 25 08 589 593 11167967 10.1046/j.1365-2230.2000.00712.x

[11] Scard C Aubert H Wargny M Martin L Barbarot S Risk of melanoma in congenital melanocytic nevi of all sizes: a systematic review J Eur Acad Dermatol Venereol 2023 37 01 32 39 36149403 10.1111/jdv.18581

